# GRNdb: decoding the gene regulatory networks in diverse human and mouse conditions

**DOI:** 10.1093/nar/gkaa995

**Published:** 2020-11-05

**Authors:** Li Fang, Yunjin Li, Lu Ma, Qiyue Xu, Fei Tan, Geng Chen

**Affiliations:** Center for Bioinformatics and Computational Biology, and Shanghai Key Laboratory of Regulatory Biology, Institute of Biomedical Sciences, School of Life Sciences, East China Normal University, Shanghai 200241, China; Center for Bioinformatics and Computational Biology, and Shanghai Key Laboratory of Regulatory Biology, Institute of Biomedical Sciences, School of Life Sciences, East China Normal University, Shanghai 200241, China; Center for Bioinformatics and Computational Biology, and Shanghai Key Laboratory of Regulatory Biology, Institute of Biomedical Sciences, School of Life Sciences, East China Normal University, Shanghai 200241, China; Center for Bioinformatics and Computational Biology, and Shanghai Key Laboratory of Regulatory Biology, Institute of Biomedical Sciences, School of Life Sciences, East China Normal University, Shanghai 200241, China; Shanghai Skin Disease Hospital, School of Medicine, Tongji University, Shanghai 200443, China; Center for Bioinformatics and Computational Biology, and Shanghai Key Laboratory of Regulatory Biology, Institute of Biomedical Sciences, School of Life Sciences, East China Normal University, Shanghai 200241, China; Shanghai Applied Protein Technology Co., Ltd. (APTBIO), Shanghai 200233, China

## Abstract

Gene regulatory networks (GRNs) formed by transcription factors (TFs) and their downstream target genes play essential roles in gene expression regulation. Moreover, GRNs can be dynamic changing across different conditions, which are crucial for understanding the underlying mechanisms of disease pathogenesis. However, no existing database provides comprehensive GRN information for various human and mouse normal tissues and diseases at the single-cell level. Based on the known TF-target relationships and the large-scale single-cell RNA-seq data collected from public databases as well as the bulk data of The Cancer Genome Atlas and the Genotype-Tissue Expression project, we systematically predicted the GRNs of 184 different physiological and pathological conditions of human and mouse involving >633 000 cells and >27 700 bulk samples. We further developed GRNdb, a freely accessible and user-friendly database (http://www.grndb.com/) for searching, comparing, browsing, visualizing, and downloading the predicted information of 77 746 GRNs, 19 687 841 TF-target pairs, and related binding motifs at single-cell/bulk resolution. GRNdb also allows users to explore the gene expression profile, correlations, and the associations between expression levels and the patient survival of diverse cancers. Overall, GRNdb provides a valuable and timely resource to the scientific community to elucidate the functions and mechanisms of gene expression regulation in various conditions.

## INTRODUCTION

Gene expression is largely controlled by upstream transcription factors (TFs) and usually exhibits spatiotemporal specificity. Specifically, each cell has a particular combination of active TFs and their downstream target genes, which form intricate gene regulatory networks (GRNs, termed regulons) (1,2). Thus, GRN profiling is important to understand the mechanisms of gene expression regulation and cellular heterogeneity (3). Dysregulation of GRNs can result in abnormal expression changes of the involved genes and contribute to the development of diseases especially cancers (4). Several databases have provided the known or predicted TF-target pairs for different organisms, such as AnimalTFDB 3.0 (5), TRRUST v2 (6) and RegNetwork (7), which mainly focus on the potential binary regulatory relationships between TFs and target genes that without the information of GRN activity and related gene expression profile (see Supplementary Table S1). Since TFs may regulate distinct sets of downstream target genes under disparate conditions (2), it is crucial to take the spatiotemporal specificity of gene expression regulation into account. However, no existing database has provided the activity of GRNs as well as the expression profiles of TFs and corresponding target genes for a variety of human and mouse conditions.

The development of bulk and single-cell RNA-sequencing (scRNA-seq) technologies and related computational methods has brought unprecedented opportunities to unravel the expression dynamics and cellular heterogeneity (8,9). In particular, scRNA-seq also enables the reconstruction of GRNs at the single-cell level and gain insights into the cell-type-specific expression regulation (2,10). For example, we recently found that TFs could regulate different gene sets across distinct subtypes of human pancreatic islets and the dynamics of GRNs is one main factor influencing the expression heterogeneity of pancreatic cells (11). Moreover, functional decay of cell-type-specific redox GRNs was revealed in primate ovarian aging, which provided potential novel biomarkers and therapeutic targets for clinical diagnosis and treatment of age-related human ovarian diseases (12). Rambow *et al.* found that GRN architecture reprogramming could play an important role in the progression and therapy resistance of melanoma (13). Additionally, enhanced expression of cell-type-specific TFs was revealed in the bronchoalveolar immune cells of the patients with Coronavirus Disease 2019 (COVID-19) through single-cell GRN analysis, suggesting that the lungs of severe COVID-19 patients had a highly proinflammatory macrophage microenvironment (14). Therefore, the activity of GRNs and the expression profiles of TFs and downstream target genes are essential to fully understand the underlying mechanisms of expression heterogeneity and the pathogenesis of diverse diseases.

GRN reconstruction on a multitude of cells/samples is time-consuming and resource-consuming, which is difficult for non-bioinformatics users to do such analysis. Furthermore, there is still lacking a database to provide the GRN activities and related gene expression profiles for a variety of physiological and pathological conditions. To address these challenges, we comprehensively inferred the GRNs and characterized the expression profiles of involved TFs and target genes based on the large-scale single-cell data of 184 human and mouse conditions as well as the bulk data of 33 cancers from The Cancer Genome Atlas (TCGA) (15) and 27 normal tissues from the Genotype-Tissue Expression (GTEx) project (16). Specifically, we developed GRNdb, a user-friendly and freely accessible database to catalog the rich information of a total of 77 746 regulons and 19 687 841 TF-target pairs, allowing users to easily explore the landscape of gene expression regulation under diverse normal tissues and diseases.

## DATA COLLECTION AND PROCESSING

### Single-cell and bulk RNA-seq data collection and processing

We collected the scRNA-seq datasets of diverse human and mouse conditions from public databases of Gene Expression Omnibus (GEO, https://www.ncbi.nlm.nih.gov/geo/) (17) and ArrayExpress (https://www.ebi.ac.uk/arrayexpress/) (18). At present, GRNdb contains 72 single-cell human conditions (332,920 cells) of various normal tissues and diseases/cancers, and 41 single-cell mouse conditions (300 150 cells) of different tissues. Moreover, we also downloaded the bulk RNA-seq expression datasets of diverse TCGA cancers (15) from UCSC Xena (https://xena.ucsc.edu/) (19) as well as the RNA-seq expression data of various normal human tissues from GTEx (https://www.gtexportal.org/home/) (16). To ensure the accuracy of gene regulatory network inference, we removed those datasets containing <30 samples. A total of 10,415 samples for 33 different cancers of TCGA and 17 333 samples of 27 distinct normal tissues of GTEx were retained. All the accession IDs of the original data for diverse human and mouse conditions are available on the ‘Statistics’ page of GRNdb.

### Cell cluster identification for scRNA-seq datasets

For the single-cell datasets that have available cell-type/cluster annotation information in the original studies, we used the known annotations of cells directly. If the scRNA-seq datasets were without available cell type/cluster annotations, we employed Seurat (version 3.1.5) (20) to define the cell clusters with the standard pipeline. Then, the maker genes with significantly enriched expression in each cell cluster of a given dataset were identified using the function of FindAllMarkers in Seurat (adjusted *P*-value < 0.05).

### Gene regulatory network reconstruction

We predicted the gene regulatory networks in various human and mouse conditions using the SCENIC pipeline (version 1.1.0.1) (10) based on the gene expression matrix of each dataset and the known TF-motif annotations. First, SCENIC employs GENIE3 (21) to detect the gene sets co-expressed with TFs, which has been demonstrated to be superior to other tools for GRN inference (22). Then, RcisTarget (10) (version 1.2.1, https://resources.aertslab.org/cistarget/) was used to infer the putative direct-binding targets of TFs based on the motif-TF annotations of cisTarget databases. Finally, the regulons were identified with the standard pipeline of SCENIC (https://github.com/aertslab/SCENIC) step-by-step. Each of the identified active regulons contains one TF and its downstream target genes. Only the best TF binding motifs that are over-represented in a given gene set were used in GRNdb. For this analysis, SCENIC utilized two different databases: (i) the database scoring the motifs in the 500 bp upstream region of the TSS and (ii) the database scoring 10 kb space around the TSS. By default, Normalized Enrichment Score (NES) > 3.0 was utilized as the threshold to define the significantly enriched motifs of corresponding TF modules in the SCENIC pipeline, which corresponds to a False Discovery Rate (FDR) between 3% and 9%.

## DATABASE CONTENT AND USAGE

### GRN landscape and statistics across various human and mouse conditions

**Figure 1.**
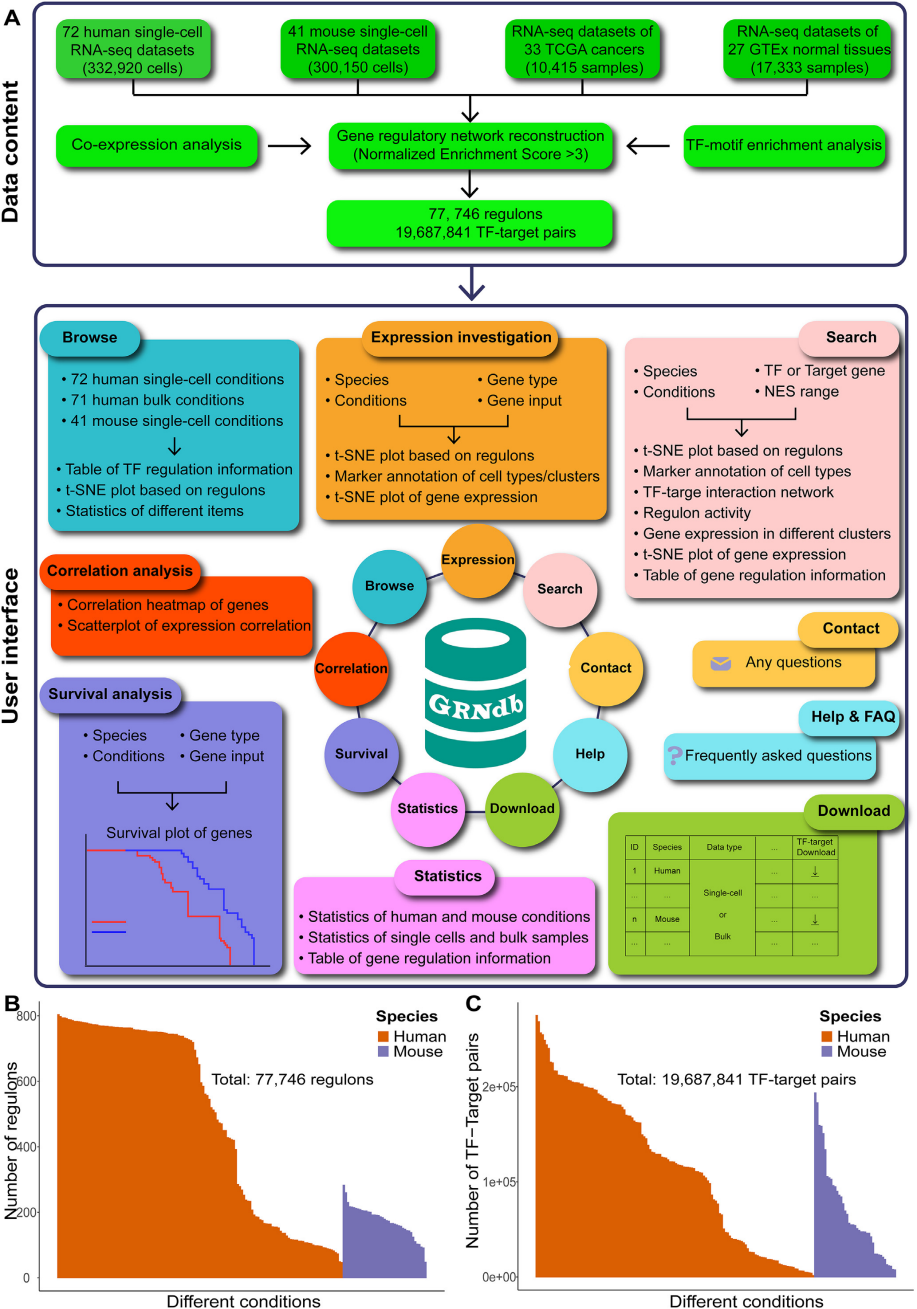
A schematic view of the GRNdb database. (**A**) Overview of data content and user interface of GRNdb. (**B**) Barplot showing the number of active regulons detected in diverse conditions of humans and mice. (**C**) Barplot displaying the number of TF-target pairs detected in various human and mouse conditions.

Currently, GRNdb provides the detailed regulon information for 143 different human physiological and pathological conditions and 41 single-cell mouse conditions of various normal tissues, involving a total of >633 070 single cells and >27 700 bulk samples (Figure 1A). Specifically, the human datasets contain 72 single-cell conditions of diverse normal tissues of adult and fetus, and the ecosystems and immune microenvironments of different tumors/diseases, as well as 71 bulk datasets covering 33 TCGA cancers and 27 normal tissues of GTEx. The robustness of GRN inference pipeline for GRNdb construction has been further validated in our recent study (11). In total, 70 651 regulons (mean: 494 and median: 585 per condition) involving 16 915 901 TF-target pairs (mean: 118 293 and median: 121 228 per condition) were detected for human, while 7095 regulons (mean: 173 and median: 179 per condition) involving 2 771 940 TF-target pairs (mean: 67 608 and median: 54 120 per tissue) were identified for mouse (Figure 1B and C). In the resulting table of database query, we provided the best TF binding motif that significantly enriched in relevant TF module for each TF-target pair (NES value > 3, equals to 0.03 < FDR < 0.09).

### GRN search, browse and visualization

**Figure 2.**
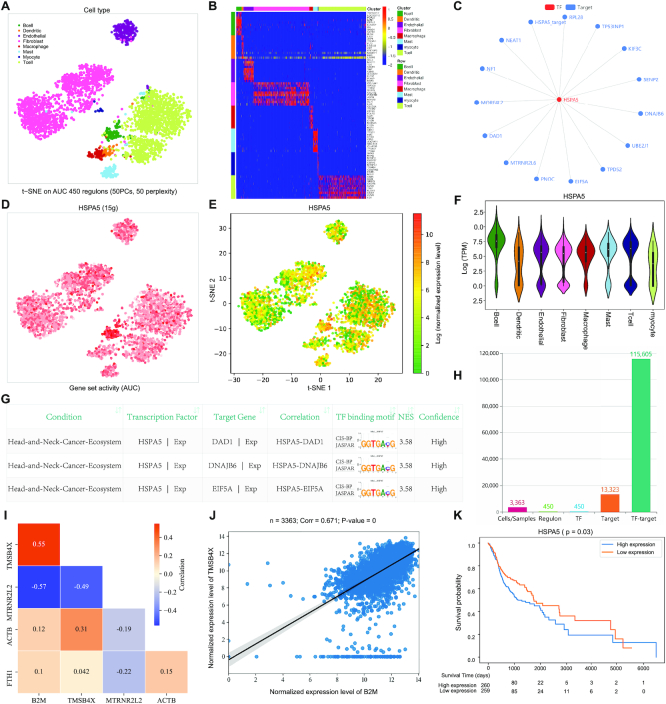
Examples of using GRNdb database. (**A**) t-SNE plot based on the regulons identified in the single-cell dataset of head and neck cancer (HNC) ecosystem. (**B**) Marker expression heatmap for different cell types of HNC. (**C**) Example showing the interaction network of TF HSPA5 and its downstream target genes detected in HNC. (**D**) Activity of the regulon formed by HSPA5 and its downstream targets in each cell of HNC. (**E**) t-SNE plot showing the expression level of HSPA5 in each cell of HNC. (**F**) Violin plot displaying the expression profile of HSPA5 in distinct cell types of HNC. (**G**) Example of the detailed regulatory information table for HSPA5 and its downstream target genes. (**H**) Statistics of the number of cells, detected regulons, TFs, targets, and TF–target pairs for HNC. (**I**) Heatmap displaying the pairwise expression correlations of B2M, TMSB4X, MTRNR2L2, ACTB and FTH1. (**J**) Scatterplot of expression correlation between B2M and TMSB4X. (**K**) Survival analysis between the expression level of TF HSPA5 and the survival of HNC patients.

On the ‘Search’ page of GRNdb, users can explore the regulatory networks of their interested TFs or target genes in diverse single-cell/bulk conditions of humans and mice. We provided three types of commonly used gene formats as input, including gene symbol, Entrez ID and Ensembl (23) gene ID. For TF querying, the returned results include the t-distributed Stochastic Neighbor Embedding (t-SNE) plot based on all the significant regulons identified in a specific condition, maker expression heatmap and annotations for different cell types/clusters (not available for bulk conditions), the regulatory network formed by the query TF and downstream target genes, regulon activity of this TF in each cell/sample, t-SNE plot of TF expression profile, violin plot of TF expression for each cell type/cluster (not available for bulk conditions), and the table of detailed information for involved TF-target pairs (Figure 2A–G). For target gene searching, it will return the results of upstream regulating TFs that were activated in a given condition. In the resulting table of gene query, all the TFs and target genes have been linked to GeneCards (24) for human and MGI database (25) for mouse, which enables users to conveniently get the detailed function and information of relevant genes. Users can dissect the expression profile of each TF and target gene in the table by simply clicking related links, which will automatically analyze the gene expression on the ‘Expression’ page. Moreover, the link of automatic expression correlation analysis for each TF-target pair is also provided. Users can also sort the column containing NES values to explore the TF-target pairs. Additionally, we annotated the TF–target pairs identified with the known annotations of cisTarget database (10) or inferred by orthology as high-confidence in GRNdb, whereas the TF–target pairs detected by motif similarity were annotated as low-confidence. Besides, the ‘Comparison’ function on the Search page enables users to conveniently compare the GRNs between any two conditions of human and mouse, which may help users to gain more insights into gene regulation.

On the ‘Browse’ page, users can browse the detailed information of all active TF–target pairs for 184 different human and mouse conditions. The browsing results of a particular condition include the t-SNE plot based on all significant regulons detected in the selected condition, the statistics barplot of the numbers for cells/samples, regulons, TFs, target genes, and TF–target pairs, as well as the detailed table of all identified TF–target pairs (Figure 2A, H and G).

### Gene expression and correlation investigation of multiple genes simultaneously

To facilitate gene expression exploration, GRNdb enables users to interrogate the expression profiles of an array of genes simultaneously on the ‘Expression’ page. The number of query genes is without limitation and the input gene format can be gene symbols, Entrez IDs or Ensembl gene IDs. This function is very useful if users want to dissect the expression profiles of many genes, which does not need to investigate the genes one by one. Expression search will return the t-SNE plot of gene expression in each cell/sample and the violin plot of gene expression in each cell type/cluster (not available for bulk conditions) (Figure 2E and F).

Moreover, ‘Expression’ page also allows the pairwise expression correlation analysis for the input gene set. This function is turned on by default, but users can disable it if they do not need to calculate the correlations. In the case of more than three input genes, a heatmap showing the pairwise Spearman's correlations of the query gene set, as well as the scatterplots of expression correlation between each pair of input genes will be displayed (Figure 2I and J). Otherwise, only one scatterplot of the Spearman's correlation between two query genes will be returned, and the correlation analysis will be inactivated if only one gene is available.

### Association analysis between gene expression and the patient survival of cancers

Considering that an important analysis in cancer studies is to check whether the expression levels of relevant genes are significantly associated with the patient survival of certain cancer, we developed the function of survival analysis for 33 different TCGA cancers on the ‘Survival’ page. For convenience, no limit is set to the number of query genes and three input formats of gene symbol, Entrez ID, and Ensembl gene ID are selectable. We implemented the Python package of lifelines (26) to conduct survival analysis and used the median expression level as the cutoff to divide the cancer patients into two different groups. A *P*-value is calculated for each gene using the Logrank test, which indicates whether the gene expression can stratify the patients into two groups with significantly distinct survival time (Figure 2K). Users can utilize the common threshold of *P*-value <0.05 to define the significance. Besides survival analysis, we also provide the TF–target regulatory network detected in related cancer for the query genes, which enables users to gain insights into the expression regulation of their interested genes.

### Download of figures, tables, and data

All the figures generated on the pages of ‘Search’, ‘Browse’, ‘Expression’ and ‘Survival’ can be downloaded by clicking the download sign on the top right corner of corresponding plot. Moreover, the resulting tables that contain the details of conditions, data types (single-cell or bulk), TFs, target genes, TF binding motifs, NES values, and confidence can also be obtained in Excel format by clicking the ‘Download’ link below the table. Additionally, on the ‘Download’ page of GRNdb, users can get the matrices containing the detailed information of all active TF–target pairs identified in diverse conditions of human and mouse. Each matrix is in plain text format with TAB separators for different columns. Users can freely utilize the plots and explore the tables they downloaded in their research.

## SYSTEM DESIGN AND IMPLEMENTATION

GRNdb was written in Python (version 3.6.8) based on the micro web framework of Flask (version 1.1.1), and its interactive interface was developed using Bootstrap (version 3.3.7) and JQuery (version 3.3.1). All the data in GRNdb are stored in a MySQL (version 8.0.20) relational database. The Echart (https://echarts.apache.org/zh/index.html) and DataTables (https://www.datatables.net/) are applied to display graphs and tables, respectively. Currently, GRNdb is deployed on a CentOS Linux server by employing Docker (version 19.03.11), an open platform for developing and running applications.

## DISCUSSION

Since genes usually exhibit spatial and temporal-specific expression, the GRNs formed by TFs and downstream target genes are also dynamically changing across different conditions or cell types/states (11,27,28). Moreover, the gene expression profiles of cells are regulated by the active regulons, which is closely related to the expression heterogeneity and phenotypes of individual cells (29). Bulk and single-cell RNA-seq technologies greatly facilitated the GRN exploration in large-scale samples/cells, providing great opportunities to characterize the underlying mechanisms of gene expression regulation and disease development (22). However, currently available TF–target regulation information for human and mouse in existing databases are generally without the information of regulon activity and expression of TFs and target genes. Thus, we developed the user-friendly database of GRNdb for users to freely access the detailed information of active regulons in diverse human and mouse conditions

At present, GRNdb provides the GRN landscape for 184 distinct physiological and pathological conditions, involving 633,070 cells and 27,748 bulk samples. A total of 70 651 regulons (16 915 901 TF-target pairs) and 7095 regulons (2 771 940 TF–target pairs) are available in GRNdb for human and mouse, respectively. All the regulations in GRNdb are predicted from the omics data, which are valuable for future experimental validations. Users can easily explore and visualize the GRNs and related gene expression profiles in various conditions. For instance, the Search page enables the GRN investigation and expression profiling of TFs and target genes (e.g. TF HSPA5 in head and neck cancer, Figure 2), while the Browse page allows examining the detailed information of all identified GRNs in each condition (Figure 2). Moreover, users can interrogate the expression profile and cancer survival of their interested genes on the pages of Expression and Survival (Figure 2), respectively. Collectively, GRNdb constitutes an abundant and valuable resource to the research community to better understand the TF–target regulations in various normal tissues and diseases. We believe GRNdb will aid users to unravel the underlying mechanisms of gene expression dynamics and disease pathogenesis. We will continue to maintain GRNdb and update it to include more human and mouse datasets.

## DATA AVAILABILITY

The GRNdb database is freely accessible for non-commercial use at http://www.grndb.com/. Users do not need to register or login to access any data available in the database.

## Supplementary Material

gkaa995_Supplemental_File
