# Supplementary material for: GRNdb: decoding the gene regulatory networks in diverse human and mouse conditions
Source: Nucleic Acids Res. 2020 Nov 5;49(D1):D97–D103. doi: 10.1093/nar/gkaa995 (PMC7779055; doi:10.1093/nar/gkaa995)
Supplement: gkaa995_Supplemental_File [file gkaa995_supplemental_file.docx]

**Supplementary Table 1. Comparison of relevant gene regulatory network databases.**

| **Items** | **GRNdb** | **AnimalTFDB 3.0** | **TRRUST v2** | **RegNetwork** |
| --- | --- | --- | --- | --- |
| Species | Human and mouse | Diverse species | Human and mouse | Human and mouse |
| Methods | Prediction based on omics data | Prediction based on TF family and classification rules | Sentence-based Text mining | Integration of selected databases |
| Visualization of GRNs | Yes | No | No | No |
| Availability of GRN activity | Yes | No | No | No |
| Visualization of gene expression | Yes | No | No | No |
| Availability of single-cell GRNs | Yes | No | No | No |
| GRN comparison between conditions | Yes | No | No | No |
| Survival analysis of cancers | Yes | No | No | No |
| TF prediction | No | Yes | No | No |
| Key regulator prediction for query genes | No | No | Yes | No |
| MicroRNA regulation | No | No | No | Yes |

Note: GRN represents gene regulatory network.
